# The Axenfeld–Rieger Syndrome Gene *FOXC1* Contributes to Left–Right Patterning

**DOI:** 10.3390/genes12020170

**Published:** 2021-01-26

**Authors:** Paul W. Chrystal, Curtis R. French, Francesca Jean, Serhiy Havrylov, Suey van Baarle, Ann-Marie Peturson, Pengfei Xu, J. Gage Crump, David B. Pilgrim, Ordan J. Lehmann, Andrew J. Waskiewicz

**Affiliations:** 1 Department of Medical Genetics, University of Alberta, Edmonton, AB T6G 2H7, Canada; pchrysta@ualberta.ca (P.W.C.); curtis.french@med.mun.ca (C.R.F.); havrylov@ualberta.ca (S.H.); vanbaar1@ualberta.ca (S.v.B.); 2 Department of Ophthalmology, University of Alberta, Edmonton, AB T6G 2H7, Canada; 3 Department of Biological Sciences, University of Alberta, Edmonton, AB T6G 2E9, Canada; fjean@ualberta.ca (F.J.); apeturso@ualberta.ca (A.-M.P.); dave.pilgrim@ualberta.ca (D.B.P.); 4 Faculty of Medicine, Memorial University of Newfoundland, St John’s, NL A1B 3V6, Canada; 5 Department of Stem Cell Biology and Regenerative Medicine, Keck School of Medicine, University of Southern California, Los Angeles, CA 90033, USA; Pengfei.Xu@med.usc.edu (P.X.); gcrump@med.usc.edu (J.G.C.); 6 Women & Children’s Health Research Institute, University of Alberta, Edmonton, AB T6G 1C9, Canada

**Keywords:** *FOXC1*, Axenfeld–Rieger syndrome, left–right patterning, zebrafish, *LEFTY*

## Abstract

Precise spatiotemporal expression of the *Nodal*-*Lefty*-*Pitx2* cascade in the lateral plate mesoderm establishes the left–right axis, which provides vital cues for correct organ formation and function. Mutations of one cascade constituent *PITX2* and, separately, the Forkhead transcription factor *FOXC1* independently cause a multi-system disorder known as Axenfeld–Rieger syndrome (ARS). Since cardiac involvement is an established ARS phenotype and because disrupted left–right patterning can cause congenital heart defects, we investigated in zebrafish whether *foxc1* contributes to organ laterality or situs. We demonstrate that CRISPR/Cas9-generated *foxc1a* and *foxc1b* mutants exhibit abnormal cardiac looping and that the prevalence of cardiac situs defects is increased in *foxc1a*^−/−^; *foxc1b*^−/−^ homozygotes. Similarly, double homozygotes exhibit isomerism of the liver and pancreas, which are key features of abnormal gut situs. Placement of the asymmetric visceral organs relative to the midline was also perturbed by mRNA overexpression of *foxc1a* and *foxc1b*. In addition, an analysis of the left–right patterning components, identified in the lateral plate mesoderm of *foxc1* mutants, reduced or abolished the expression of the *NODAL* antagonist *lefty2*. Together, these data reveal a novel contribution from *foxc1* to left–right patterning, demonstrating that this role is sensitive to *foxc1* gene dosage, and provide a plausible mechanism for the incidence of congenital heart defects in Axenfeld–Rieger syndrome patients.

## 1. Introduction

The establishment of left–right asymmetry represents a fundamental step in embryonic development. Despite substantial progress elucidating a proportion of the core players, the mechanisms remain incompletely defined. Consequently, syndromes where organs are aberrantly positioned are of particular interest to geneticists and developmental biologists. In humans, the heart’s normal anatomical position is left of midline, with a larger left ventricle designed for systemic circulation. The right lung has three lobes, while the left lung has two lobes and contains an indentation, the cardiac notch, allowing space for the heart. Furthermore, the stomach and liver are positioned left and right of the midline, respectively. This normal arrangement is called situs solitus, while the complete reversal of normal organ situs (termed situs inversus) is surprisingly well tolerated [1]. Far more deleterious are partial situs defects, collectively known as heterotaxy [2], characterized by mis-patterning of visceral organs along the left–right axis. These are associated with congenital diseases of the heart, lungs, spleen, stomach, and liver [3,4,5] that may be particularly challenging to treat. Intriguingly, many heterotaxy-associated genes also cause isolated congenital heart defects (CHDs) [6], suggesting that a proportion of idiopathic CHDs may reflect unrecognized situs defects [2,7,8,9].

In vertebrates, the breaking of left–right symmetry is established around a structure known as the left–right organizer (LRO). In mouse, zebrafish, frog, and humans, asymmetric fluid flow, generated by motile monocilia projecting into the extracellular fluid of the LRO, leads to asymmetric gene expression patterns around the LRO [10,11,12]. Consequently, loss of flow or of the motile cilia results in situs defects in animal models and humans [12,13,14,15]. In many vertebrates, the output of the LRO first manifests as a decreased expression of *DAND5* (also known as *charon* in zebrafish or *Cerl2* in mouse), which normally represses the transforming growth factor beta (TGF-β) family member *Nodal*, a secreted morphogen that is transiently expressed on the left side of the embryo [16,17,18]. Nodal upregulates its own transcription as well as transcription of the homeobox domain transcription factor *Pitx2* [19]. Despite asymmetric *Nodal* expression lasting only a matter of hours, murine *Pitx2* expression persists in the left lung and cardiac tissue throughout organogenesis and into adulthood [19,20,21,22,23]. Pitx2 plays a critical role in the establishment of left–right asymmetry, and homozygous murine mutants display pronounced phenotypes. For example, at E12.5, murine *Pitx2* homozygotes display cardiac situs defects and right pulmonary isomerism (identical lobar anatomy of the left and right lungs) [22], while second heart field-specific *Pitx2* mutations cause severe cardiac outflow tract defects [24]. In patients, heterozygous *PITX2* mutations cause Axenfeld–Rieger syndrome (ARS); however, despite multi-organ involvement, organ situs defects have not been observed [25,26,27,28]. This likely reflects patients’ heterozygous variants, compared with homozygous deletion of *Pitx2*, that can be achieved either globally or in the secondary heart field of murine models [22,24].

Precise control of left–right patterning relies on the establishment of a signaling barrier to separate left- and right-specific gene expression programs. Critical components of the midline barrier are the TGF-β family members *Lefty1/2* [29,30,31,32] that are expressed in the midline and on the left side and diffuse to the midline and right side to inhibit Nodal signaling. Although both genes are expressed in the same region, the increased diffusion of Lefty proteins (relative to Nodal) limits Nodal target gene activation on the right side of the embryo [10,33]. Consequently, mice with a deletion in the *Lefty2* enhancer that is activated by Nodal display left isomerism [34] while variants in *LEFTY1/2* are associated with congenital heart defects [35]. Illustrating significant additional complexities in the control of left–right patterning, pathways initiated on the right side of the embryo have also been shown to contribute to organ laterality, as demonstrated by bone morphogenetic protein (BMP)-dependent activation of Prrx1a in cardiac laterality and the role of hyaluronan in determining midgut laterality [36,37].

Axenfeld–Rieger syndrome (ARS) is an autosomal dominant condition caused by mutation and copy number variation of *PITX2* and *FOXC1* [26,28,38,39,40,41,42,43]. The ARS phenotypic spectrum includes ocular anterior segment dysgenesis, early-onset glaucoma, craniofacial dysmorphism, cerebral small vessel disease, cerebellar vermis hypoplasia, and hydrocephalus [26,44,45,46,47]. Despite no reported association with laterality defects, congenital heart defects are present in ARS, and these include atrial and ventricular septal defects, valve stenosis, and persistent truncus arteriosus [26,48,49,50,51,52], particularly associated with *FOXC1* mutation [25,26,27,53]. Murine *Foxc1* mutants also exhibit CHDs, but no alteration of L–R patterning has been reported [54,55,56].

To test the hypothesis that Foxc1 is a regulator of L–R patterning, we mutated the two zebrafish paralogs via clustered regularly interspaced short palindromic repeats (CRISPR)/Cas9 editing. In addition to replicating many of the ARS-associated phenotypes observed in human *FOXC1* mutation, this strategy yielded evidence for a requirement for zebrafish *foxc1a/b* in establishing cardiac and gut laterality. Analysis of these zebrafish mutants also established that the expression of *lefty2* was altered. Our data thus provide the first evidence of a contribution by Foxc1 to left–right patterning.

## 2. Materials and Methods

### 2.1. Zebrafish Lines and Husbandry

Zebrafish lines were kept in accordance with the University of Alberta’s Animal Care and Use Committee guidelines. Animal care protocols were approved by the University of Alberta Biosciences Animal Care Committee with protocol number 00000082. Experiments were performed in the AB line, and *foxc1a*^ua1017^ and *foxc1b*^ua1018^ were generated and maintained on the AB background. The Tg(sox10:GFP)^ba4^ line was used for examination of the craniofacial deformities caused by *foxc1* mutation [57]. Zebrafish embryos were raised at 28.5 °C or 33 °C in E3 media or E3/0.2 mM 1-phenyl-2thiourea (PTU) from 22 h post fertilization (hpf) onwards to prevent pigmentation [58]. Standard staging of embryos was conducted as in Kimmel et al. 1995 [59]. *foxc1a*^el542^ and *foxc1b*^el620^ have been previously described [60].

### 2.2. Bioinformatics

ENSEMBL sequences (human ENSG00000054598 and ENSG00000176692; mouse ENSMUST00000062292 and ENSMUST00000054691, xenopus ENSXETG00000000594 and ENSXETG00000016387, and zebrafish ENSDARG00000091481 and ENSDARG00000055398) were aligned with Clustal Omega for sequence conservation analysis. The synteny of human and zebrafish *FOXC1* orthologs were investigated in ENSEMBL using the “region in detail” options to explore the chromosomal regions surrounding each gene, and schematics were generated in Corel Draw.

### 2.3. LRO Cilia Length Measurements

Embryos raised at 33 °C were collected at the 10 ss and fixed overnight at 4 °C in 4% paraformaldehyde (PFA). Storage at −20 °C in 100% methanol was usually performed for up to two weeks. Once transferred into phosphate buffered saline—tween 20 (PBS-T), embryos were dechorionated and blocked in 10% heat-inactivated goat serum and 1% bovine serum albumin for 1 h while rocking. Primary antibody (monoclonal anti-tubulin, acetylated—Sigma, St. Louis, MO, USA, T6793) at 1:1000 dilution was performed overnight at 4 °C. Embryos were washed (3 × 30 min PBS-T), and then, secondary antibody (goat anti-mouse Alexa Fluor^®^ 555—Abcam, Cambridge, UK, ab150114) at 1:2000 and a nuclear counterstain (TO-PRO™-3 Iodide ThermoFisher Scientific, Waltham, MA, USA, T3605) at 1:2000 was performed for 2 h before 3 × 15 min PBS-T washes and a final PBS-T wash overnight at 4 °C while rocking. Embryos were transferred into 70% glycerol via gradient (30%, 50%, and 70%), and the posterior region of the embryo including the LRO was excised with forceps and mounted in a slide viewing chamber and covered with a coverslip. The remainder of the embryo was processed for gDNA extraction. Imaging stacks through the whole LRO were performed on an LSM 700 confocal microscope (Zeiss, Oberkochen, Germany) using a 40× oil lens with a 1 µm Z-interval. Maximum intensity projections were generated, and axonemal length for each cilium was calculated in FIJI, with the average cilia length per LRO per embryo being used for statistical analysis.

### 2.4. Bead Tracking

Left–right organizer function was assayed as described in [61]. Embryos at the 11–12 hpf were dechorionated and embedded in a few drops of 1% low melting point agarose before the LRO was injected with approximately 0.5 nL of Fluoresbrite Polychromatic Red 0.5 Micron Microspheres (Polysciences, Warrington, FL, USA, #19507). Bead flow was recorded for 10 s at 20× differential interference contrast on an Axioskop 2 microscope (Zeiss, Oberkochen, Germany) using QCapture Plus. FIJI software using the Manual Tracker plug-in was used to produce the bead projections and record metrics.

### 2.5. In Situ Hybridization

*In situ* hybridization was performed essentially as described in [62]. Briefly, digoxigenin-labelled probes were synthesized from linear DNA templates using RNA polymerase and digoxigenin-UTP kits (Roche, Basel, Switzerland) and were purified using SigmaSpin columns (Sigma, St. Louis, MO, USA). Fixed embryos were permeabilized with proteinase K treatment for 3 min (18–20 hpf) or 15 min (48 hpf), re-fixed with 4% PFA for 20 min, and hybridized with 1:200 RNA probes overnight at 65 °C. High stringency washes in 0.2× and 0.1× SSC/0.1% Tween 20 were carried out at 65 °C for 20 min each, and blocking was performed for 1 h in 2% sheep serum and 2 mg/mL bovine serum albumin. Anti-digoxigenin-AP antibody (Roche, Basel, Switzerland) at 1:5000 dilution was used to detect probe hybridization, and NBT/BCIP (Roche, Basel, Switzerland) coloration reactions were performed at 33 °C until the signal was saturated. *In situs* were imaged by dissection light microscopy, and then, the tissue was genotyped. The probes used were *myl7* [63]; *foxa3* [64]; pitx2c and elvol6 [65]; foxc1a and foxc1b [66]; spaw fwd: ATGCAGCCGGTCATAGC, rev: TCAATGACAGCCGCACTC; *lefty1* fwd: ATATTCTGACACGACACGTC, rev: CTGAAATATTGTCCATTGC; and *lefty2* fwd: ATCAAGTACTCGGACACC, rev: GGAGTCCCATAACTGTG.

### 2.6. Light Microscopy

Live imaging was performed on PTU-treated embryos anesthetized in 0.6 mM Tricaine (Sigma, St. Louis, MO, USA, Cat.# A-5040) as per [67]. Both live imaging and *in situ* processed embryos were imaged on a 1% agarose coated dishes using a SZX12 light microscope (Olympus, Tokyo, Japan) with QCapture Suite Plus. White balance and brightness/contrast editing were performed in Adobe^®^ Photoshop^®^ and were performed consistently between all genotypes and conditions.

### 2.7. mRNA Overexpression and Morpholino Microinjection

Capped mRNA was generated from a linear DNA template in a pCS2+ vector using the mMessage mMachine (ThermoFisher Scientific, Waltham, MA, USA) kit, purified using TRIzol™ (ThermoFisher Scientific, Waltham, MA, USA) and microinjected into the one-cell stage embryo at doses of 5, 15, and 75 pg. The highest 75 pg dose was used for all subsequent experiments unless otherwise stated. All RNA technical replicates were performed on a single day, injecting *foxc1* transcripts and control RNA at the same dose into the same clutch of embryos. Morpholino oligonucleotides *foxc1a*—CCTGCATGACTGCTCTCCAAAACGG—and *foxc1b*—GCATCGTACCCCTTTCTTCGGTACA—were previously reported [46].

### 2.8. Organ Situs Scoring

Cardiac situs scoring was performed by light microscopy on live 48 hpf embryos. While still in the chorion, embryos were anesthetized and rolled with fine watchmaker’s forceps so they could be viewed ventrally. The sequential atrial–ventricular heart contractions allowed for the cardiac situs to be easily scored based on looping of the ventricle in comparison to the atrium. To assess gut situs, *in situ* hybridization on 48 hpf embryos was performed using a *foxa3* probe, and once developed, the images of each embryo were recorded. Scoring of heart and gut situs were performed in a masked manner, and subsequently, the tissue was processed for gDNA extraction.

### 2.9. Rhodamine-Conjugated Dextran Injections

Hydrocephalus was examined in zebrafish embryos as in Lowery et al. [68]. Briefly, Texas Red Dextran (10,000 MW) (Life Technologies D1828) was dissolved to 5 mg/mL in Danieau Buffer (17.4 mM NaCl, 0.21 mM KCl, 0.12 mM MgSO_4_·7H_2_O, 0.18 mM Ca(NO_3_)_2_·4H_2_O, and 1.5 mM HEPES). The embryos were anesthetized in 0.1 mg/mL Tricaine (Sigma, St. Louis, MO, USA), embedded in 1.5% low-melting point agarose (Millipore, Burlington, VT, USA, A9414), and injected with 4 nL Dextran into the hindbrain ventricle. The embryos were immediately recovered from agarose and imaged no longer than 5 min after injection on a dissection microscope by brightfield and fluorescence. The images were overlayed in Adobe Photoshop to produce figures.

### 2.10. Genotyping

Embryonic tissue was dissociated in 50 mM NaOH as in [69] and then diluted 1:10 before being used as template for PCR. Both foxc1 mutations were resolved via standard PCR genotyping: *foxc1a* fwd: TTCTTCGCCAGCTGTACG, rev: AATAACTTTGGTCGCTGC and *foxc1b* fwd: CCGTGTCTAGCCAAAGC, rev: TCGGATGAGTTTTGGATG. Gel electrophoresis was used to resolve the wildtype (WT) and mutant bands under the following conditions: *foxc1a*—sodium borate buffer, 3% agarose, 300 V for 1 h (as in [70]), *foxc1b*—Tris-acetate-EDTA buffer, 2% agarose, 150 V for 1 h.

### 2.11. Statistical Analysis

Situs scoring of the heart and gut from *foxc1* mutants was categorical data so Fisher’s exact test was performed comparing normal and abnormal frequency between mutants and the WT control. The embryos from at least five clutches of embryos were scored and pooled for our analysis. For both mRNA overexpression and morpholino knockdown, the percentage of situs abnormalities was calculated per technical replicate (a clutch of embryos injected with the mRNA or MO). The mean percentage situs was compared via ANOVA with Dunnett *post hoc* test to determine which categories were significantly different. All graphs and statistics were performed in GraphPad Prism 8.

## 3. Results

### 3.1. Generation of foxc1a and foxc1b Zebrafish Mutants

**Figure 1:**
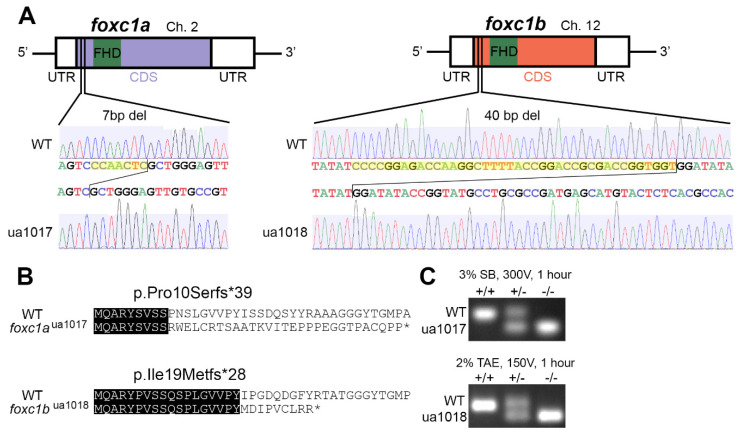
Allelic consequence of *foxc1*-targeted CRISPR/Cas9 mutagenesis: (**A**) a schematic representation of *foxc1a*^ua1017^ and *foxc1b*^ua1018^ mutations, with 7 and 40 base pair deletions (yellow highlighting) upstream of the DNA-binding Forkhead domain; (**B**) the predicted sequences of the first 40 amino acids translated from wildtype (WT) and mutant proteins with sequence identity (black highlighting), where the *foxc1a*^ua1017^ allele produces a truncated 39-residue protein with loss of sequence homology from amino acid 10 and the *foxc1b*^ua1018^ allele produces a truncated 28-residue protein with loss of sequence homology from amino acid 19; and (**C**) PCR genotyping from the gDNA template resolving the respective deletions in *foxc1a* and *foxc1b*. (FHD, Forkhead domain; UTR, untranslated region; CDS, coding sequence.).

Zebrafish possess two orthologs of human *FOXC1* (*foxc1a* and *foxc1b*) but lack a *FOXC2* ortholog [71]. While it has been suggested that zebrafish *foxc1b* may represent a functional ortholog of human *FOXC2* [66], the amino acid sequences of Foxc1a and Foxc1b are more similar to human FOXC1 (identities: 74% and 68%, respectively) than FOXC2 (55% and 53%) (Table S1), with equivalent results when comparing the DNA-binding Forkhead domains (FOXC1: 97% and 97%, and FOXC2: 93% and 93%). Since the zebrafish paralogs possess conserved synteny with human *FOXC1* (Figure S1) as well as similar protein sequences and overlapping expression [66,71], we anticipated compensatory activity with loss of a single paralog. To address this, we generated mutations in both *foxc1a* and *foxc1b* using CRISPR/Cas9 mutagenesis. The *foxc1a*^ua1017^ allele is a 7 nucleotide deletion that is predicted to cause a premature stop codon after 39 amino acids (c.29_35del; p.Pro10Serfs*39). The *foxc1b*^ua1018^ allele is a 40 nucleotide deletion that results in a premature stop codon after 28 amino acids (c.57_96del; p.Ile19Metfs*28) (Figure 1A–C). These are hereafter referred to as *foxc1a*^−/−^ and *foxc1b*^−/−^.

The novel *foxc1a*^−/−^ and *foxc1b*^−/−^ mutations lie upstream of the Forkhead DNA-binding domain and result in frameshifts that are predicted to result in loss of >90% of the encoded protein (Figure 1B). Consistent with the known mechanisms of non-sense mediated decay [72,73,74], we do not observe degradation of mRNA in either *foxc1a*^−/−^ or *foxc1b*^−/−^ homozygotes (Figure S2). Although the multiple methionine residues located between these mutations and the highly conserved Forkhead domain have the potential to function as alternative start sites (Figure S3), both mutations occur in the N-terminal transactivation domain where even small deletions profoundly reduce *FOXC1* activity [75]. Furthermore, our novel mutations overlap previously published *foxc1* null mutations (el542, el543, and el620 [60]) and phenocopy loss-of-function mutations in the Forkhead box DNA-binding domain [76]. For these reasons, we believe that the novel *foxc1* mutations provide good models for *foxc1* loss-of-function.

### 3.2. foxc1a Single and Double Mutants Display Gross Developmental Defects

**Figure 2:**
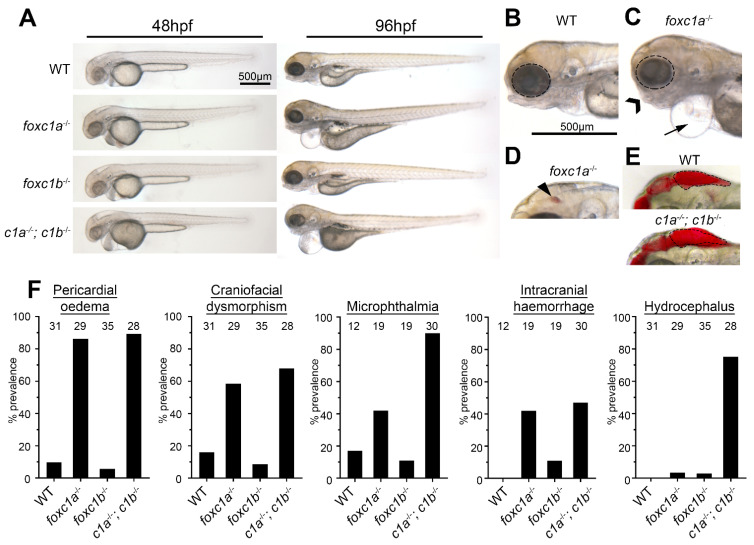
*Foxc1* mutants exhibit multiple developmental defects. (**A**) *foxc1* single mutants are largely indistinguishable from WT (wildtype) controls at 48 hpf (left panels), whereas *foxc1a*^−/−^; *foxc1b*^−/−^ double homozygotes display hydrocephalus and oedema. By 96 hpf, no changes are observed in *foxc1b*^−/−^ homozygotes, however; *foxc1a*^−/−^ homozygotes and *foxc1a*^−/−;^ *foxc1b*^−/−^ double homozygotes display pericardial oedema (compare **B** and **C**, as highlighted by the arrow), microphthalmia (dotted circle), and craniofacial dysmorphism (chevron). At this stage, a subset of mutants also displayed intracranial hemorrhage (arrowhead in panel **D**), with *foxc1a*^−/−^ homozygotes having greater frequency than *foxc1b*^−/−^ homozygotes (42% vs. 11%, respectively), and (**E**) *foxc1a*^−/−^; *foxc1b*^−/−^ double homozygotes present with hydrocephalus. (**F**) Quantification reveals that these defects are incompletely penetrant and generally more prevalent in double than single homozygotes (the number of embryos analyzed is shown above each bar). In the case of hydrocephalus, only the double homozygotes display an appreciable frequency of this phenotype.

*foxc1a*^+/−^ zebrafish are viable and fertile as heterozygotes and generate Mendelian ratios of larvae when incrossed. However, *foxc1a*^−/−^ homozygotes do not live beyond 7 days post fertilization (dpf) and display obvious developmental defects by 96 h post fertilization (hpf; Figure 2A–F). Blood flow is observed until 72 hpf when there is pericardial oedema in 86% of embryos (Figure 2C) that becomes more severe over time. Zebrafish *foxc1a*^−/−^ homozygotes also display microphthalmia (Figure 2B,C and Figure S4) and intracranial hemorrhage (Figure 2D) at 72 hpf. *foxc1a*^−/−^ homozygotes display craniofacial dysmorphism (59%; Figure S5), which has been reported previously [60].

Zebrafish *foxc1b*^−/−^ homozygotes are viable as larvae, survive to adulthood, and are fertile with no observable phenotype (results consistent with other studies [60,66]). Incrossing two *foxc1b*^−/−^ homozygous adults to generate maternal zygotic *foxc1b*^−/−^ homozygote offspring also generated viable embryos indistinguishable from wildtype controls. Prior research on the zebrafish *foxc1b*^−/−^ determined that it is a loss-of-function allele [77]. To investigate the potential for genetic compensation, we next examined the expression patterns of *foxc1a* and *foxc1b* during early development. Both *foxc1* mRNAs are maternally inherited as evidenced by *in situ* hybridization patterns at the 1 and 8 cell stages (Figure S6). During epiboly, the *foxc1* expression patterns overlapped in the presumptive segmental plate and in the segmental plate and head mesenchyme during segmentation stages, albeit more weakly for *foxc1b*. Finally, at the 18 somite stage (ss), *foxc1* paralog expression was greatest in the periocular mesoderm, pharyngeal arch, and segmental plate. *foxc1a* expression was also observed in the sprouting intersegmental vessels.

Considering the substantial overlap in expression patterns, we generated a double *foxc1a/foxc1b* mutant line to determine if the phenotype became more severe. Both double heterozygotes (*foxc1a*^+/−^; *foxc1b*^+/−^) and fish with 3 mutant alleles (*foxc1a*^+/−^; *foxc1b*^−/−^) were viable as adults and were fertile. Crossing two double heterozygotes generated double mutant homozygote larvae (*foxc1a*^−/−^; *foxc1b*^−/−^) at expected Mendelian ratios, although developmental deformities were severe, especially with regards to microphthalmia and hydrocephalus (Figure 2E,F). Eighty-nine percent of *foxc1a*^−/−^; *foxc1b*^−/−^ double homozygotes displayed pericardial oedema at 72 hpf, which suggest poor cardiac and/or pronephric duct function, at which time, 72 hpf blood flow had also ceased. Craniofacial dysmorphism was observed in 68% of embryos and was more severe than single *foxc1a*^−/−^ homozygotes. Additionally, 75% of *foxc1a*^−/−^; *foxc1b*^−/−^ double homozygotes displayed hydrocephalus, which was absent in single mutants (Figure 2E,F). These data demonstrate that a loss of zebrafish *foxc1a/b* paralogs is generally more severe than single homozygosity and supports the hypothesis of genetic buffering by the two paralogs.

### 3.3. Alterations to foxc1 Gene Dosage Cause Visceral Organ Situs Defects

**Figure 3:**
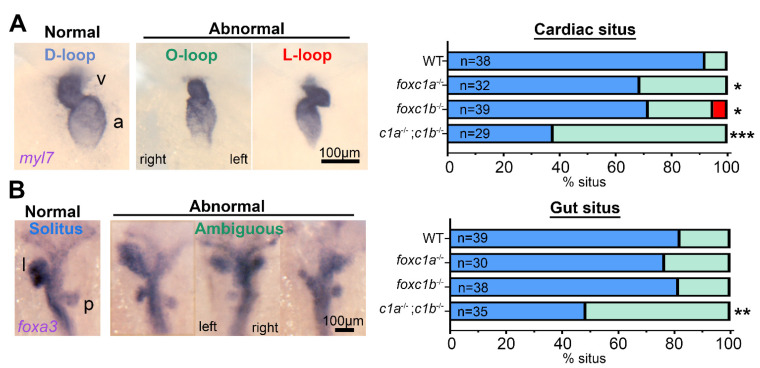
Decreased dosages of *foxc1* result in multi-organ situs defects. (**A**) *In situ* hybridization with *myl7* at 48 hpf revealed aberrant cardiac situs (O-loop (green); L-loop (red)) compared with normal D-loop (blue). The prevalence of aberrant situs is increased in *foxc1a*, *foxc1b*, and double homozygotes (* *p* = 0.016, * 0.036, *** < 0.001 respectively, Fisher’s exact test) when compared to WT siblings. (**B**) At the same stage, the normal arrangement (solitus, blue) of the gut is left-sided liver (l) and right-sided pancreas (p); however, the incidence of abnormal (ambiguous, green) gut situs was significantly greater in double homozygotes (** *p* = 0.003, Fisher’s exact test).

Due to the loss of blood flow observed from 72 hpf in *foxc1a* homozygotes, we next examined cardiac development in more detail. Loss of *foxc1* resulted in significantly fewer embryos with normal D-looped hearts and increased the prevalence of abnormal O-looped (unlooped) and L-looped (situs inversus) hearts (Figure 3A). Homozygous *foxc1a* mutant hearts failed to loop in 31% of those examined (*n* = 32, *p* = 0.016), while homozygous *foxc1b* mutant embryos displayed an elevated prevalence of abnormal cardiac looping (23% O-loop and 5% L-looped hearts; *n* = 39, *p* = 0.036). These findings are consistent with the overlapping expression patterns of the two genes and support a conserved function in patterning of cardiac situs. These results are directly supported by an analysis of cardiac looping in double *foxc1a*^−/−^; *foxc1b*^−/−^ homozygotes, where 62% of *foxc1a*^−/−^; *foxc1b*^−/−^ embryos have abnormal O-loops (*n* = 29, *p* < 0.001). 

To investigate whether the situs defects were cardiac specific or systemic, gut situs was assessed using the liver and pancreas expression domain of *foxa3*. Although neither *foxc1a*^−/−^ nor *foxc1b*^−/−^ homozygotes exhibited altered gut situs, this was present in 51% of *foxc1a*^−/−^; *foxc1b*^−/−^ double homozygous embryos. In these, the most common phenotype was isomerism of the liver and / or pancreas (Figure 3B), and overall, these results suggest that cardiac situs is more sensitive to loss of *foxc1* than gut situs.

**Figure 4:**
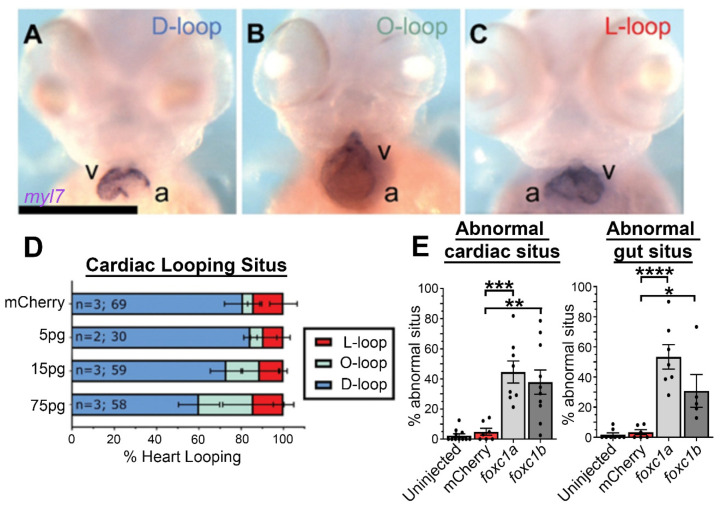
*Foxc1a* mRNA overexpression causes cardiac situs defects in a dose-dependent manner: (**A**–**C**) *myl7 in situ* hybridization of 75 pg *foxc1a* mRNA-injected embryos at 48 hpf, with representative images of the three cardiac looping morphologies provided (v, ventricle; a, atrium); (**D**) quantification of cardiac situs in control and *foxc1a* mRNA-injected embryos revealing an increasing prevalence of anomalous cardiac looping with increasing amounts of *foxc1a* mRNA; and (**E**) quantification of embryo situs defects in embryos injected with 75 pg of *foxc1a* mRNA. Statistical significance was apparent in comparisons between *foxc1a/b* and *mCherry* controls (cardiac: *p* = 0.0002 and 0.0012; gut: *p* < 0.0001 and 0.0205. mCherry vs. *foxc1a* and *foxc1b* respectively. One-way ANOVA and Dunnett’s *post hoc* test. * *p* < 0.05, ** *p* < 0.01, *** *p* < 0.001, **** *p* < 0.0001).

Because *FOXC1* function is exquisitely sensitive to gene dosage, with both gene duplication and deletion causing disease [25,41,42,78], we examined whether mRNA overexpression of *foxc1* induces cardiac situs defects (Figure 4). An injection of 75 pg of either *foxc1a* or *foxc1b* mRNA resulted in divergence from normal D-looped hearts observed in 45% of *foxc1a* and 38% of *foxc1b* mRNA-injected embryos, with the prevalence greatly increased from the mCherry mRNA control (Figure 4A–C). Such cardiac situs defects were observed in a dose-dependent manner (Figure 4D), and hydrocephalus commonly arose at 72 hpf in embryos when either *foxc1a* or *foxc1b* was overexpressed (Figure S7). Gut situs was similarly affected (Figure 4E), with a comparable prevalence of abnormal gut situs observed (*foxc1a* overexpression: 53%, *foxc1b* 31%, mCherry control 3%, *p* = 0.02).

Although these data support a requirement for *foxc1* in normal organ situs determination in zebrafish and were supported by morpholino knockdown (Figure S8), this finding ran counter to conventional understanding of the role of Foxc1. Therefore, to validate these results, we assayed independently generated zebrafish alleles [60] and demonstrate that these *foxc1a*^−/−^; *foxc1b*^−/−^ double homozygotes exhibit similar cardiac and gut situs defects (Figure S9).

### 3.4. Loss of foxc1a/b Does Not Disrupt LRO Fluid Flow

**Figure 5:**
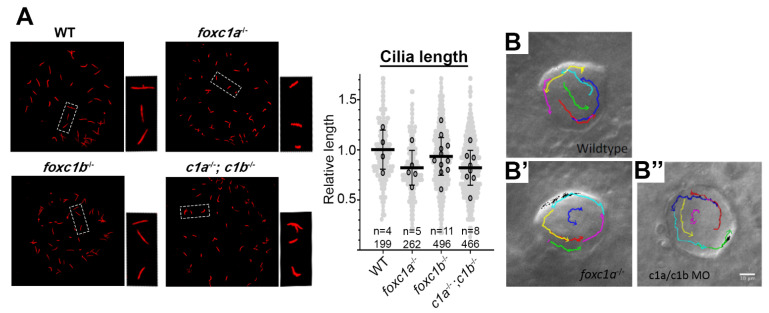
Loss of *foxc1* does not significantly change cilia length or left–right organizer flow. (**A**) Acetylated α-tubulin immunostaining of left–right organizer (LRO) cilia revealed that average cilia length was not significantly altered in *foxc1* mutants (*foxc1a*^−/−^, 82%; *foxc1b*^−/−^, 93%; *foxc1a*^−/−^; *foxc1b*^−/−^, 82% of WT length; *p* = 0.286, ANOVA; 4–11 embryos imaged per condition, all cilia per condition shown in light grey). Tracking of fluorescent bead flow in the LRO revealed unchanged counterclockwise flow between WT (**B**), *foxc1a*^−/−^ homozygotes (**B’**), and *foxc1a*/*foxc1b* morphants (**B’’**).

The left–right organizer (LRO) is a transient organelle that has been described in multiple vertebrate species, including zebrafish (Kupffer’s vesicle), and is required for left–right axis patterning. *foxj1*-dependent motile cilia within the LRO generate leftward fluid flow to initiate left–right asymmetric gene expression of the lateral plate mesoderm. Accordingly, we next examined LRO cilia in single and double *foxc1* zebrafish mutants to determine if alterations were present. Alterations to the axonemal length of *foxc1a*^−/−^, *foxc1b*^−/−^, and double *foxc1a*^−/−^; *foxc1b*^−/−^ homozygotes (Figure 5A; 82%, 93%, and 82% of WT respectively) did not reach statistical significance (*p* = 0.279). In order to determine if the loss of *foxc1* in zebrafish instead impacted the biological function of LRO cilia, nodal flow was measured via fluorescent bead tracking. These experiments in *foxc1a*^+/−^ heterozygous incrosses revealed comparable ciliary-driven counterclockwise flow in wildtype embryos, *foxc1a*^−/−^ homozygotes, and *foxc1a/b* morphants (Figure 5B,B’,B’’). Together, these data suggest that loss of *foxc1a/b* does not appreciably alter LRO ciliary function. These findings are consistent with the absence of observable *foxc1a* or *foxc1b* expressions in the dorsal forerunner cells or LRO during gastrulation and segmentation stages, respectively (Figure S6).

### 3.5. foxc1 Mutants Have Loss of lefty2 Expression in the Lateral Plate Mesoderm

**Figure 6:**
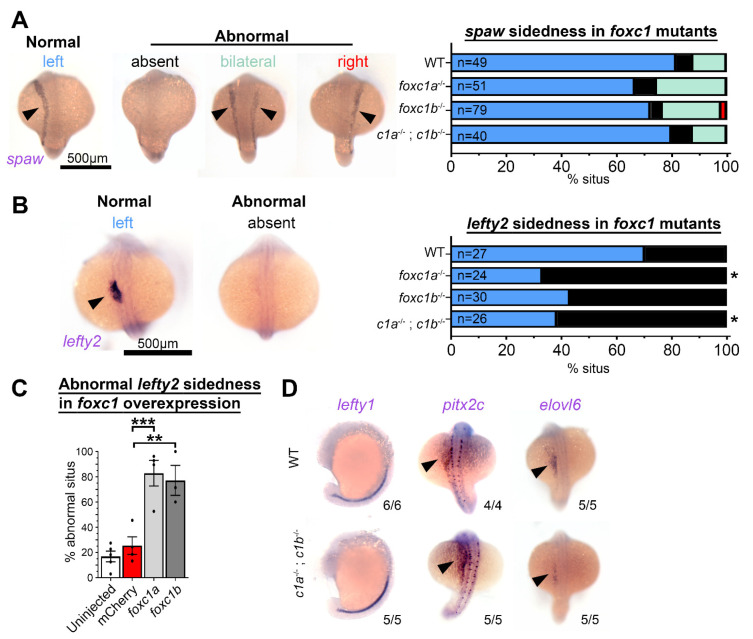
*Foxc1* mutants display loss of *lefty2* expression independent of changes to other left–right patterning genes. (**A**) *In situ* hybridization with *southpaw* (spaw) revealed no difference in the prevalence of normal left-sided expression (normal = blue, absent = black, bilateral = green, and right = red) in *foxc1* mutants compared to controls (*p* > 0.1, Fisher’s exact test). (**B**) Conversely, *lefty2* expression was significantly altered in *foxc1* mutants. Normal left-sided *lefty2* expression was absent more frequently in *foxc1a*^−/−^ and double *foxc1a*^−/−^; *foxc1b*^−/−^ homozygotes (*p* = 0.012 and 0.028, respectively) and trended to be absent in *foxc1b*^−/−^ homozygotes without reaching statistical significance (*p* = 0.061, Fisher’s exact test) (normal = blue and absent = black). (**C**) *lefty2* expression was significantly abnormal, with the overexpression of *foxc1a* or *foxc1b* (*p* = 0.0007 and 0.0030, respectively, ANOVA and Dunnett’s Test). (**D**) Analysis of the additional left–right patterning genes *lefty1*, *pitx2c*, and *elvol6* revealed no differences between WT, and *foxc1a*^−/−^; *foxc1b*^−/−^ double homozygotes. (* *p* < 0.05, ** *p* < 0.01, *** *p* < 0.001).

Since no changes at the level of the LRO were resolved, we next examined L–R axis determinants downstream of the LRO. The early L–R patterning gene *southpaw* (*spaw*), equivalent to mammalian *Nodal*, did not show altered expression patterns in *foxc1* mutants (Figure 6A). In contrast, the Nodal antagonist *lefty2* was altered in the lateral plate mesoderm (Figure 6B): 33% of *foxc1a*^−/−^ and 38% of *foxc1a*^−/−^; *foxc1b*^−/−^ double homozygotes exhibited normal left-sided expression (Figure 6B). *foxc1b*^−/−^ single mutants trended towards altered *lefty2* expression, with only 43% having normal left-sided expression, although they did not reach statistical significance (*p* = 0.061). This inconsistency in the roles of *foxc1a* and *foxc1b* in controlling *lefty2* expression may represent a divergence of protein function between the paralogs or alternatively be due to a lack of statistical power of our analysis. However, a consistent trend was observed with the independently generated *foxc1a*^−/−^; *foxc1b*^−/−^ double homozygotes (Figure S9) [60]. These data suggest that the Foxc1 paralogs regulate expression of *lefty2* during establishment of the left–right axis, and consistently, the expression domains of both paralogs overlap the primitive heart fields (Figure S6), where *lefty2* is also expressed [79]. Furthermore, overexpression of both paralogs induced the loss of left-sided *lefty2* expression in approximately 83% of *foxc1a* and 77% of *foxc1b* embryos compared to 25% of mCherry control 25% (*p* = 0.0007 and 0.003, respectively) (Figure 6C and Figure S10). Together, these data demonstrate that increased and decreased *foxc1* expression perturb *lefty2* patterning.

This finding of *lefty2* mis-expression led us to examine other genes required for the establishment of the left–right axis. *Lefty1* is a *spaw* antagonist closely related to Lefty2 but is expressed in the embryonic midline. *pitx2c* is asymmetrically expressed in the left LPM and lies downstream of *spaw*. No changes to the expression of either gene were resolved in double homozygotes at the 22 ss or 18 ss respectively (Figure 6D). Finally, *in situ* analysis of *elovl6*, an enzyme asymmetrically expressed in lateral plate mesoderm that is responsive to LRO function [65,80], revealed no changes in *foxc1a*^−/−^; *foxc1b*^−/−^ double homozygotes at the 18 ss. These results suggest that Foxc1 regulates *lefty2* rather than induces loss or randomization of all left–right patterning gene expressions, as seen when LRO function is perturbed [81]. Loss of *foxc1* may therefore be sufficient to perturb organ situs in a partially penetrant manner.

## 4. Discussion

Our findings expand the role of the Forkhead box transcription factor Foxc1 in a new direction by establishing a novel contribution to patterning of the vertebrate left–right body axis. Through CRISPR-Cas9 mutation of the zebrafish *foxc1a* and *foxc1b* paralogs, we demonstrate that loss of a single paralog induces cardiac situs defects. Consistently, mutation of both paralogs results in more extensive alterations to left–right patterning, with the involvement of multiple visceral organs evident from cardiac and gut situs defects in double homozygous *foxc1* mutants. To our knowledge, this is the first report of single *foxc1b*^−/−^ homozygotes possessing a phenotype. We also demonstrate that, unlike other *FOX* paralogs [82,83,84], a disruption of ciliogenesis was not the mechanism of action for organ situs in the *foxc1* mutants. From a mechanistic perspective, we show that *foxc1* mutation disrupts *lefty2* expression in the lateral plate mesoderm, findings recapitulated in independently generated *foxc1a*^−/−^; *foxc1b*^−/−^ mutants. This perturbation of a key antagonist of left-sided identity, taken together with our other data, reveals a hitherto unappreciated role of *foxc1* in regulating organ laterality and illustrates how analyses of *foxc1a/b* mutant zebrafish can inform studies of patients and murine loss-of-function ARS models.

Our novel zebrafish *foxc1* mutations recapitulate many phenotypes observed in Axenfeld–Rieger syndrome (ARS) patients and animal models thereof [46,47,85]. By mutating both zebrafish orthologs of human *FOXC1*, we show that *foxc1a/b* homozygotes display craniofacial dysmorphism, cardiac defects, and intracranial hemorrhage. These phenotypes correlate closely with clinical data, where up to 43% of patients exhibit craniofacial anomalies [25], 11% exhibit congenital heart defects [27], and 73% exhibit white matter hyperintensities [46]. The absence of most phenotypes in *foxc1b*^−/−^ homozygotes could suggest considerable genetic compensation and/or residual activity of the protein. Firstly, previous studies whereby *foxc1b* was knocked down via morpholino oligonucleotides failed to resolve a phenotype despite *foxc1a* knockdown resulting in microphthalmia, somitic delay, and intracranial hemorrhage [46,66,86]. Furthermore, *foxc1b* mutants have also been described without resolving a phenotype [60,76]. Our findings demonstrate that *foxc1b* is required for cardiac situs in isolation. Secondly, similar human mutations upstream of the Forkhead box DNA-binding domain are loss-of-function alleles that cause Axenfeld–Rieger syndrome [38,42,87]. Considering the significantly overlapping expression patterns of the *foxc1* paralogs, it is unsurprising that genetic buffering is observed; this is evidenced by the presence of gut situs defects and an almost complete penetrance of hydrocephalus in *foxc1a*^−/−^; *foxc1b*^−/−^ double homozygotes, which is lacking in both our *foxc1a* single mutants and those previously published [76]. Hydrocephalus specifically is a key phenotype of the murine *Foxc1*^−/−^ mutant “*congenital hydrocephalus*” and an occasional finding in patients with heterozygous *FOXC1* mutation or deletion [27,38].

The Forkhead box transcription factor family is evolutionarily conserved from yeast to humans and comprises more than 45 members in mammals, each containing an 80–100 amino acid winged-helix DNA-binding domain. Extensive links to development and disease have been established for many members of this family, and intriguingly, three *FOX* genes are recognized regulators of L–R patterning. *Foxj1* is expressed in the highly ciliated choroid plexus, lung epithelium, oviduct, and testis [88], and *Foxj1* knockout mice display loss of motile cilia and abrogation of left–right patterning. *Foxj1* is thus recognized as a key regulator of motile ciliogenesis [83]. *Foxa2* plays a role in promoting the expression of *Pkd1l1* and is essential for forming the left–right organizer [89], while *Foxh1* (also known as *Fast*) functions as a Smad co-factor downstream of Nodal signaling [90]. Two additional findings led us to examine a role for Foxc1 in visceral organ situs. First, congenital heart defects (frequently associated with heterotaxy) are present in heterozygous patients and *Foxc1* knockout mice. Second, Pitx2, a protein that may directly bind Foxc1 [91], and also causes ARS in humans, is a critical regulator of left–right patterning. Our data demonstrate that *foxc1* mutation induces situs defects of visceral organs, with two mutant copies required for cardiac situs defects, while four are needed for anomalous gut situs. One explanation for the appearance of gut situs defects only in double homozygotes is genetic buffering. Indeed, the expression patterns of the *foxc1* paralogs largely overlap and some tissues tolerate the loss of Foxc1a as evidenced by the appearance of hydrocephalus only in *foxc1a*^−/−^; *foxc1b*^−/−^ homozygotes. Recapitulation of these phenotypes with independently generated mutants and mRNA overexpression of either *foxc1a* or *foxc1b* demonstrates that increases and decreases in the levels of these *foxc1* paralogs impacts the situs of multiple visceral organs.

The establishment of left–right patterning can be divided into three separable phases: the creation of a ciliated epithelium that drives leftward fluid flow; the initiation of left-specific *Nodal-Pitx2* gene expression; and finally, the induction of a midline barrier that blocks Nodal activity from signaling on the right side. Our studies on Foxc1 demonstrate normal cilia-mediated flow in the LRO and proper initiation of left-specific *spaw* and *pitx2c*, consistent with Foxc1 playing a later role in regulating left–right axis formation. Indeed, when characterizing left–right isomerism defects in *foxc1a/b* mutants, we note that phenotypes are not consistent with a randomization of organ situs, as would have been expected from an early role in establishing left-specific gene expression. Furthermore, we find markedly perturbed *lefty2* expression in *foxc1a/b* mutants. Lefty proteins, which are known antagonists of Nodal (Spaw) signaling, have significantly greater rates of diffusion than the Nodal proteins that they antagonize [33]. This leads to the prevailing model for how Lefty functions as a midline barrier to block extracellular Nodal from reaching the right side of the embryo. Consistent with the observed heterotaxic effects on the left–right placement of heart, liver, and pancreas, we conclude that Foxc1 proteins play a key role in the regulation of *lefty* gene expression and are thus likely components of antagonistic control of the Nodal morphogen.

In this manuscript, we present the first evidence of *foxc1* playing a role in left–right patterning of the lateral plate mesoderm and in controlling organ situs. These data have significant implications for understanding the etiology of Axenfeld–Rieger syndrome associated congenital heart defects and strengthens the case for cardiac screening in patients diagnosed with ARS. Our findings should also encourage a reexamination of organ situs in other Foxc1 models, since situs defects may be subtle and only partially penetrant. Since Foxc1 is the fourth Forkhead gene to participate in left–right patterning, this result emphasizes the gene family’s importance in the control of organ situs and will encourage future studies to determine how multiple Forkhead family members evolved seemingly distinct roles in the establishment of the left–right body axis.

## Data Availability

Data is contained within the article or Supplementary Materials.

## Supplementary Materials

The following are available online at https://www.mdpi.com/2073-4425/12/2/170/s1, Figure S1: Syntenic analysis of the foxc1 paralogs. Figure S2: foxc1 mutations foxc1aua1017 and foxc1bua1018 does not induce nonsense mediated decay. Figure S3: The ua1017/ua1018 alleles are nonsense mutations overlapping previously published foxc1 null alleles. Figure S4: foxc1a mutants are microphthalmic. Figure S5: foxc1a mutants have defects in craniofacial cartilage. Figure S6: foxc1 expression during left-right patterning. Figure S7: Overexpression of foxc1a or foxc1b causes hydrocephalus. Figure S8: Morpholino knockdown recapitulates mutant situs phenotypes. Figure S9: Organ situs and left-right patterning defect trends in distinct alleles of *foxc1a*^−/−^; *foxc1b*^−/−^. Figure S10: foxc1a and foxc1b overexpression result in absent *lefty2* expression. Table S1: Both zebrafish Foxc1 protein sequences are orthologous to human FOXC1.
