# Supplementary material for: The Axenfeld–Rieger Syndrome Gene FOXC1 Contributes to Left–Right Patterning
Source: Genes (Basel). 2021 Jan 26;12(2):170. doi: 10.3390/genes12020170 (PMC7912076; doi:10.3390/genes12020170)
Supplement: Supplementary file 1 [file genes-12-00170-s001.pdf]

Supplementary Figures

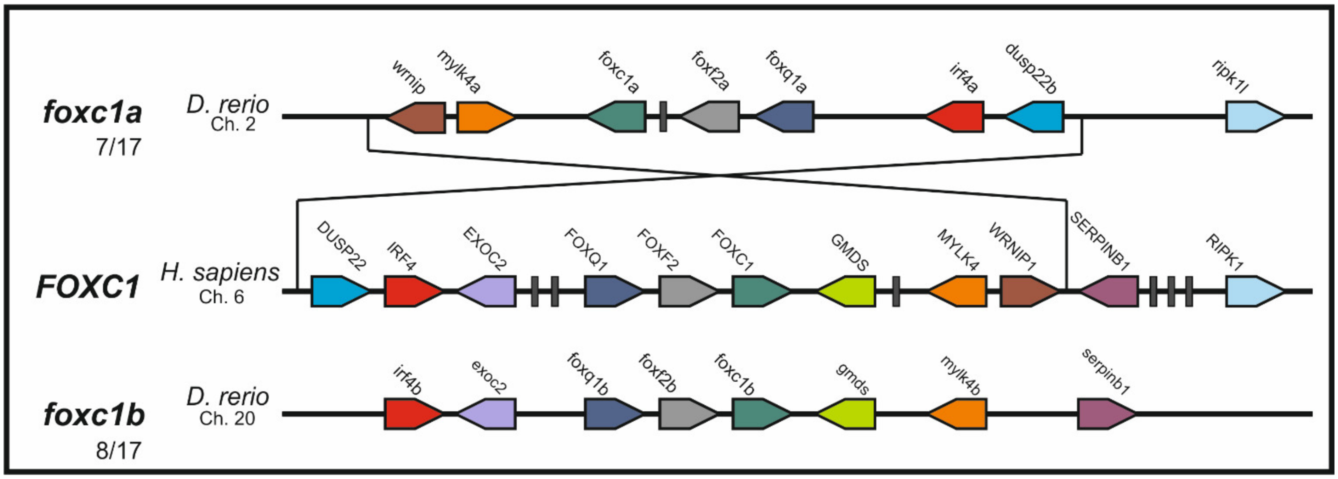

Figure S1 Syntenic analysis of the *foxc1* paralogs. Schematic representation of the chromosomal regions surrounding human *FOXC1*, and zebrafish paralogs. *foxc1a* shares 7 of 17, and *foxc1b* 8 of 17, orthologous genes with the corresponding human chromosomal region and both zebrafish chromosomes maintain the conserved *foxq1*, *foxf2*, *foxc1* triplet.

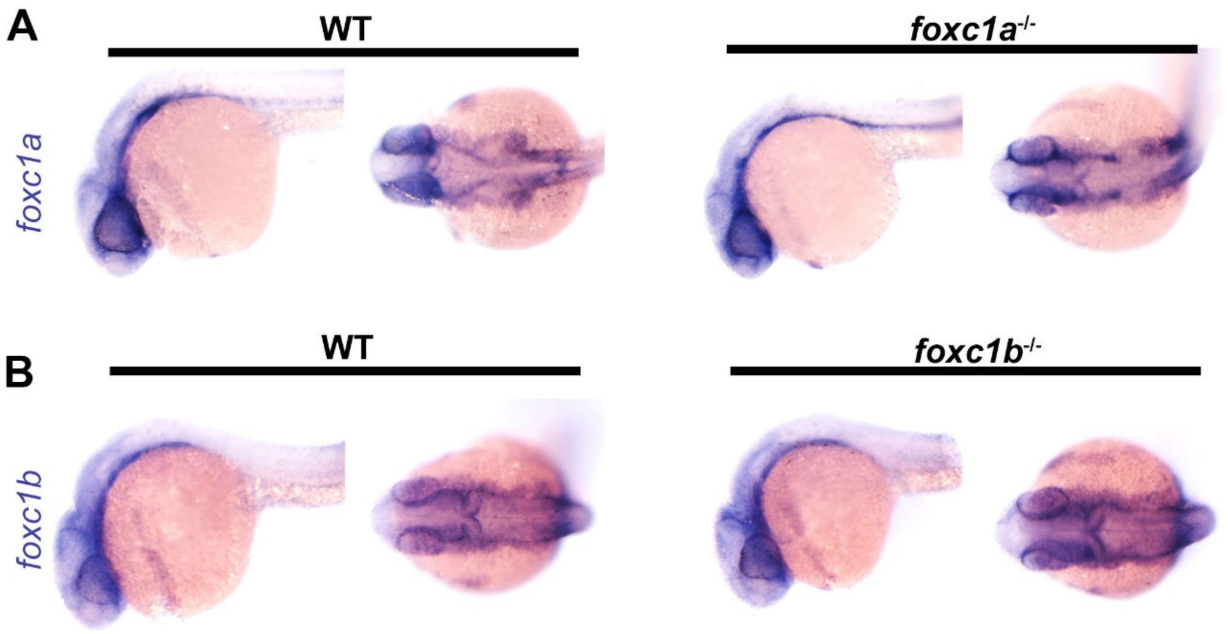

Figure S2 *foxc1* mutations *foxc1a*^{ua1017} and *foxc1b*^{ua1018} does not induce nonsense mediated decay. *In situ* hybridisation at 24 hpf using *foxc1a* (**A**) and *foxc1b* (**B**) specific probes demonstrates that mutant mRNA is not degraded in *foxc1a*^{ua1017} or *foxc1b*^{ua1018} homozygotes respectively.

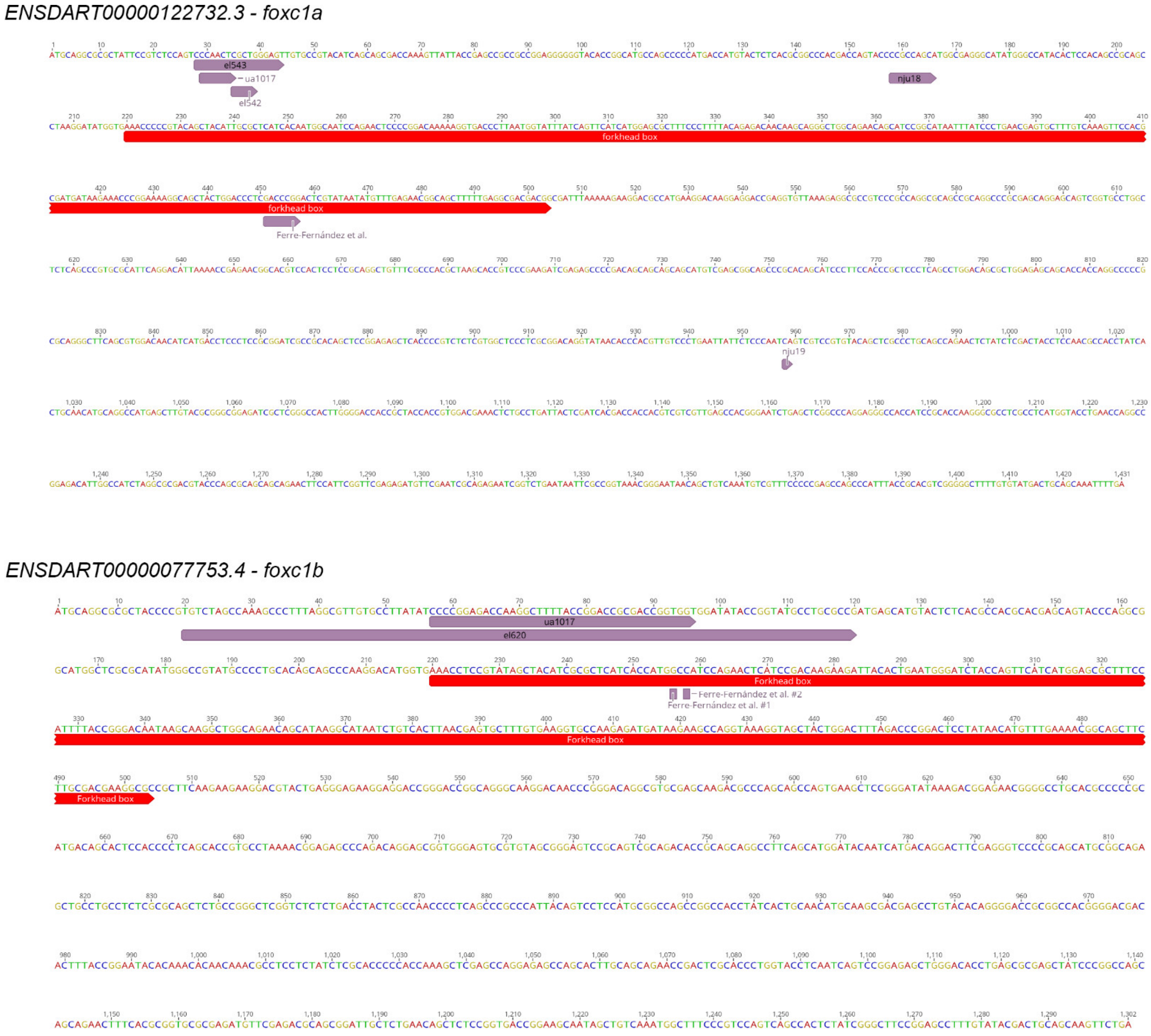

Figure S3 The ua1017 / ua1018 alleles are nonsense mutations overlapping previously published foxc1 null alleles. Sequences of full length *foxc1a* (ENSDARG00000091481) and *foxc1b* (ENSDARG00000055398) cDNAs highlighting the location of the ua1017 / ua1018 alleles, previously published *foxc1* alleles and the region coding the forkhead box DNA-binding domain. Underlined methionines represent possible alternative START sites located downstream of the ua1017 and ua1018 mutations and upstream of the forkhead box DNA-binding domain. el542, el543, el620 [1], nju18, nju19 [2] and all unnamed alleles [3].

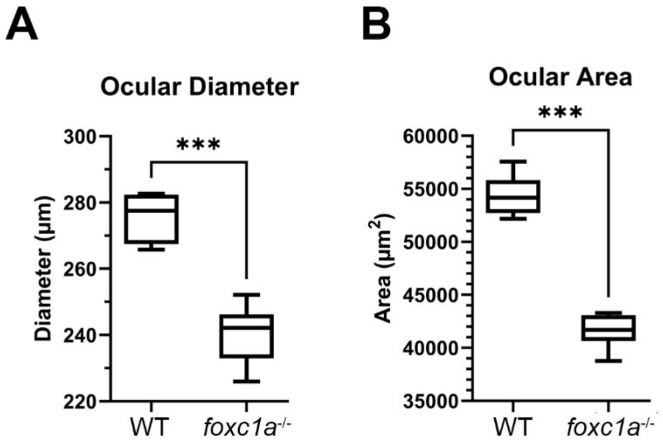

Figure S4 ***foxc1a*** **mutants are microphthalmic.** Measuremens of ocular diameter (**A**) and ocular area (**B**) of *foxc1a*-/- homozygotes at 96 hpf. N=7, Mann-Whitney U test, *** *P*<0.001.

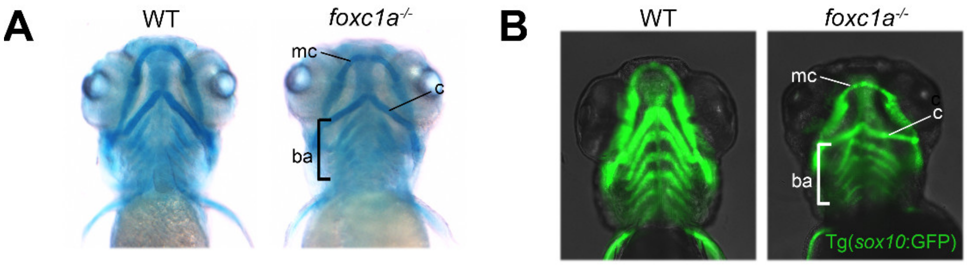

Figure S5 ***foxc1a*** **mutants have defects in craniofacial cartilage.** (**A**) Alcian blue staining of the craniofacial skeleton at 5 dpf. *foxc1a*$^{-/-}$ homozygotes display craniofacial dysmorphism compared to WT controls. (**B**) The Tg(sox10:GFP) transgene remains visible at 5 dpf in the craniofacial skeleton and reveals disorganisation of the brachial arches (ba), as well as a significant reduction in the size of Meckel's (mc) and the Ceratohyal cartilages (c)

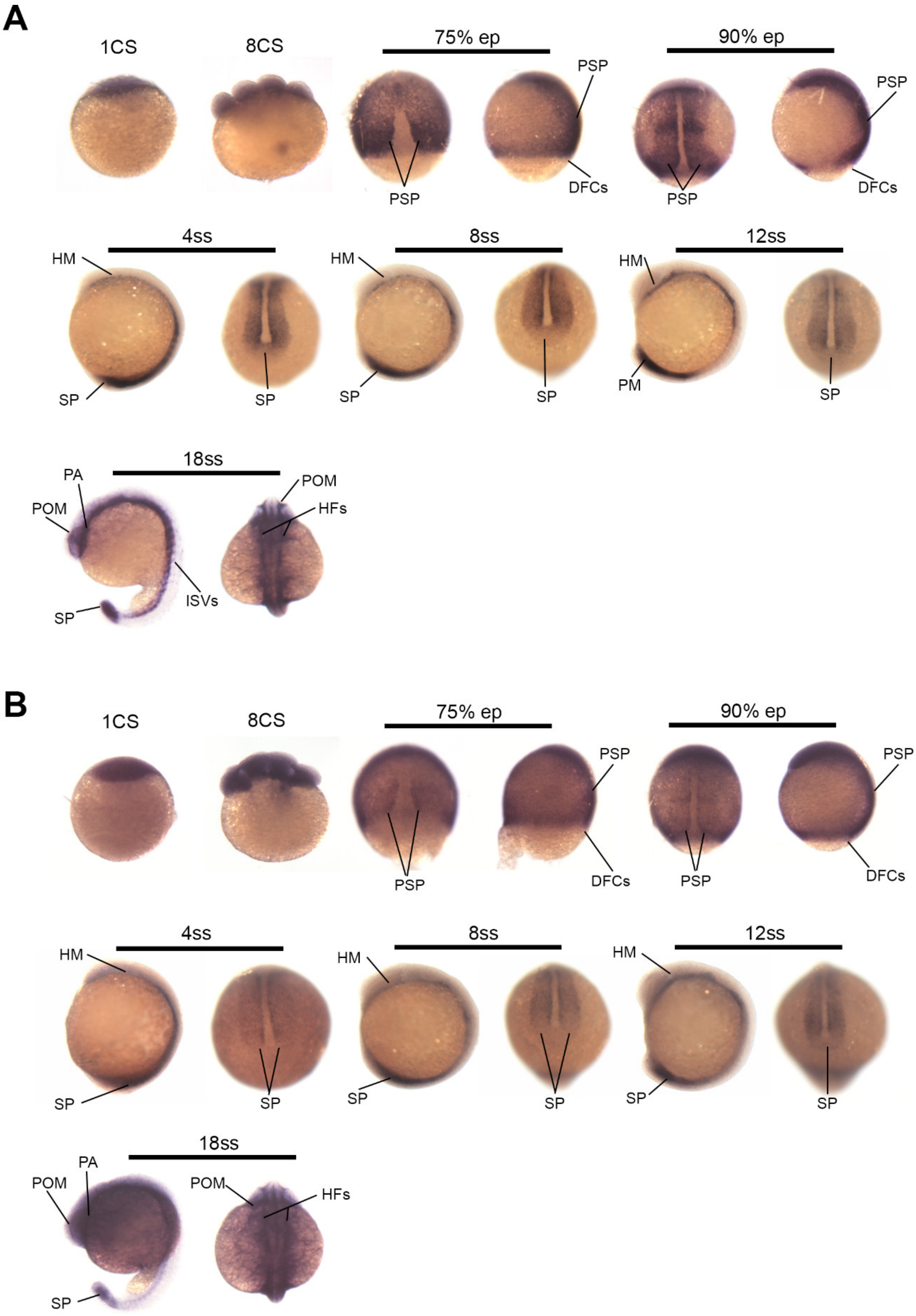

Figure S6 ***foxc1*** **expression during left-right patterning.** (**A**) Maternal deposition of *foxc1a* is apparent at both the 1 and 8 cell stage. During the 75 and 95% epiboly stages, *foxc1a* is expressed in the presumptive segmental plate (PSP) but absent from the dorsal forerunner cells (DFCs). During segmentation stages *foxc1a* is expressed in the

head mesenchyme (HM) and segmental plate (SP). At the 18ss *foxc1a* is expressed in the segmental plate as well as the axial vessels (AV), periocular mesenchyme (POM) and bilateral heart fields (HFs). (**B**) *foxc1b* has broadly overlapping expression patterns to *foxc1a*. *foxc1b* is maternally deposited and then weakly expressed in the presumptive segmental plate during epiboly but is absent from the dorsal forerunner cells. During segmentation, *foxc1b* is expressed in the head mesenchyme and segmental plate, and by the 18ss is also expressed in the periocular mesenchyme and heart fields.

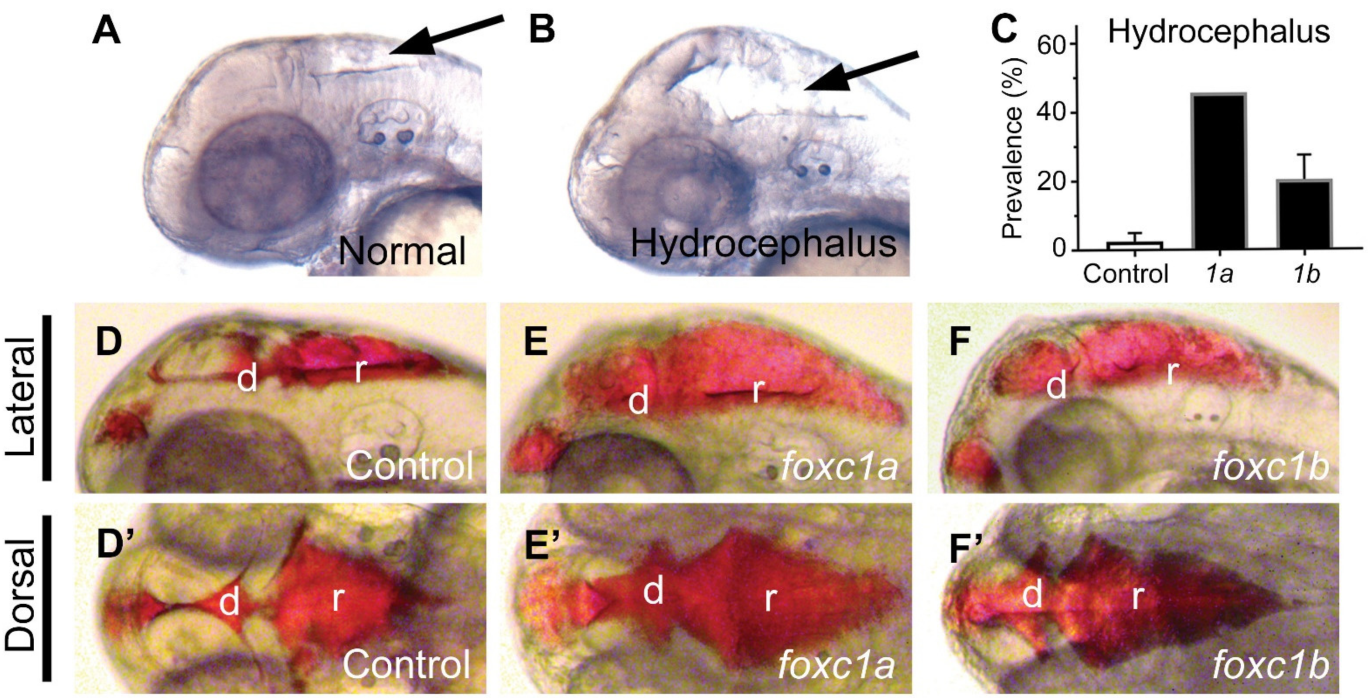

Figure S7 Overexpression of foxc1a or foxc1b causes hydrocephalus. Compared to 75 pg mCherry injected controls (**A**), 75 pg of *foxc1a* or *foxc1b* mRNA result in development of hydrocephalus (arrow) at 72 hpf (**B**) at a prevalence of 46% and 20%, respectively (**C**). Microinjection of rhodamine-conjugated dextran into the diencephalic ventricle allows for visualisation of the entire cerebral ventricle system (**D-F′**). In both *foxc1a* and *foxc1b* OE there is expansion of the diencephalic (d) and rhombencephalic (r) ventricles. Panels show hydrocephalic cerebral ventricles from both lateral (**D-F**) and dorsal (**D′-F′**) aspects.

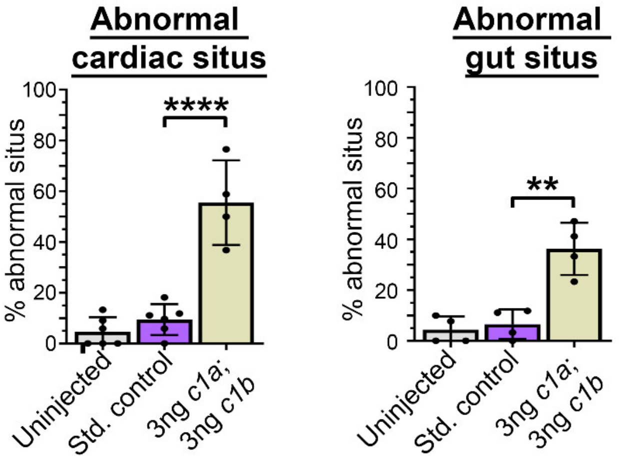

Figure S8 Morpholino knockdown recapitulates mutant situs phenotypes. Abnormal organ situs was significantly more frequent when *foxc1a* and *foxc1b* were knocked down via morpholino oligonucleotide (cardiac: *P*<0.0001; gut, *P*= 0.0003, ANOVA and Dunnett test).

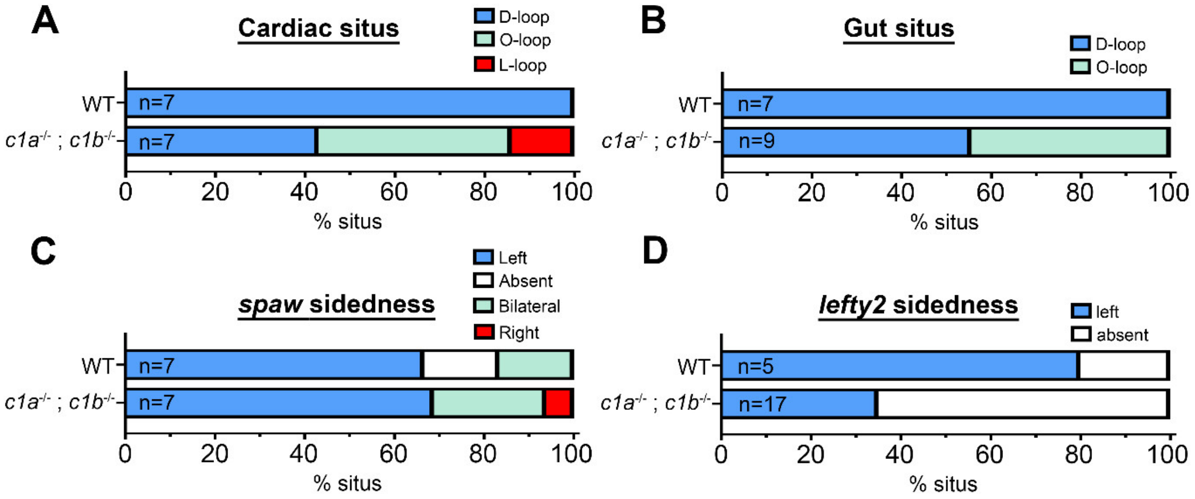

Figure S9 Organ situs and left-right patterning defect trends in distinct alleles of *foxc1a*$^{-/-}$;*foxc1b*$^{-/-}$. Examination of *foxc1a*^{el542}; *foxc1b*^{el620} double homozygous mutants [4] at 48 hpf revealed a trend for cardiac situs (**A**) and gut situs (**B**) defects in *foxc1a*$^{-/-}$; *foxc1b*$^{-/-}$ homozygotes (*P*=0.0699, 0.0885 respectively). At 18 hpf sidedness of *southpaw* (**C**) was comparable between WT and double mutants (*P*>0.99), however *lefty2* expression (**D**) trended towards more frequent absence in double mutants compared to WT (*P*= 0.1353, Fisher's exact test).

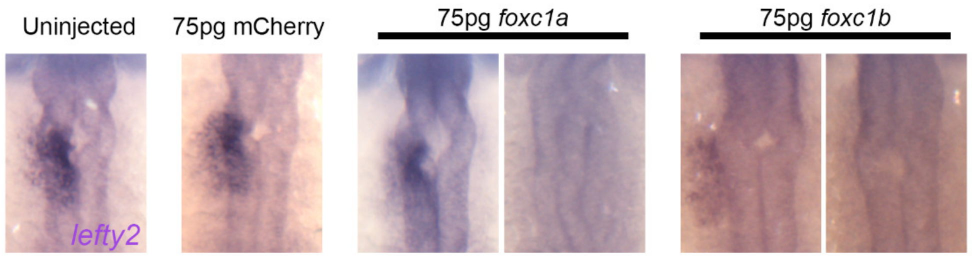

Figure S10 *foxc1a* and *foxc1b* overexpression result in absent *lefty2* expression. Representative images of *lefty2 in situ* hybridisation expression patterns at the 22ss. Shown are examples of the four conditions examined: uninjected and mCherry injected controls, and in *foxc1a* and *foxc1b* overexpressing embryos.

Gene	% amino acid identity whole protein		% amino acid identity FKH domain	
	Foxc1a	Foxc1b	Foxc1a	Foxc1b
D. rerio Foxc1a	100	-	100	-
D. rerio Foxc1b	72	100	98	100
H. sapiens FOXC1	74	68	97	97
H. sapiens FOXC2	55	53	93	93
M. musculus Foxc1	75	68	97	97
M. musculus Foxc2	54	52	93	93
X. tropicalis Foxc1	80	71	97	97
X. tropicalis Foxc2	56	55	95	95

Table S1 Both zebrafish Foxc1 protein sequences are orthologous to human FOXC1. The zebrafish Foxc1a and Foxc1b protein sequences were aligned against each other (grey) and the human, mouse and frog FOXC1 (blue) and FOXC2 (white) sequences. Irrespective of whether the whole protein or the *Forkhead* domain (FKH) were compared, the zebrafish paralogs were more similar in sequence to FOXC1 orthologs than FOXC2.

1. Xu, P.F.; Balczerski, B.; Ciozda, A.; Louie, K.; Oralova, V.; Huysseune, A.; Crump, J.G. Fox proteins are modular competency factors for facial cartilage and tooth specification. *Development* **2018**, *145*, doi:UNSP dev165498
10.1242/dev.165498.
2. Li, J.; Yue, Y.; Dong, X.; Jia, W.; Li, K.; Liang, D.; Dong, Z.; Wang, X.; Nan, X.; Zhang, Q., et al. Zebrafish foxc1a plays a crucial role in early somitogenesis by restricting the expression of aldh1a2 directly. *J Biol Chem* **2015**, *290*, 10216-10228, doi:10.1074/jbc.M114.612572.
3. Ferre-Fernández, J.J.; Sorokina, E.A.; Thompson, S.; Collery, R.F.; Nordquist, E.; Lincoln, J.; Semina, E.V. Disruption of foxc1 genes in zebrafish results in dosage-dependent phenotypes overlapping Axenfeld-Rieger syndrome. *Hum Mol Genet* **2020**, *29*, 2723–2735, doi:10.1093/hmg/ddaa163.
4. Xu, P.; Balczerski, B.; Ciozda, A.; Louie, K.; Oralova, V.; Huysseune, A.; Crump, J.G. Fox proteins are modular competency factors for facial cartilage and tooth specification. *Development* **2018**, *145*, doi:10.1242/dev.165498.
